# Mechanisms of angiogenic incompetence in Hutchinson–Gilford progeria via downregulation of endothelial NOS

**DOI:** 10.1111/acel.13388

**Published:** 2021-06-04

**Authors:** Yantenew G. Gete, Luke W. Koblan, Xiaojing Mao, Mason Trappio, Bhushan Mahadik, John P. Fisher, David R. Liu, Kan Cao

**Affiliations:** ^1^ Department of Cell Biology and Molecular Genetics University of Maryland College Park MD USA; ^2^ Merkin Institute of Transformative Technologies in Healthcare Broad Institute of Harvard and MIT Cambridge MA USA; ^3^ Department of Chemistry and Chemical Biology Harvard University Cambridge MA USA; ^4^ Howard Hughes Medical Institute Harvard University Cambridge MA USA; ^5^ Fischell Department of Bioengineering University of Maryland College Park MD USA

**Keywords:** ABE, aging, endothelial cells, eNOS, progeria

## Abstract

Hutchinson–Gilford progeria syndrome (HGPS) is a rare genetic disorder with features of accelerated aging. The majority of HGPS cases are caused by a *de novo* point mutation in the *LMNA* gene (c.1824C>T; p.G608G) resulting in progerin, a toxic lamin A protein variant. Children with HGPS typically die from coronary artery diseases or strokes at an average age of 14.6 years. Endothelial dysfunction is a known driver of cardiovascular pathogenesis; however, it is currently unknown how progerin antagonizes normal angiogenic function in HGPS. Here, we use human iPSC‐derived endothelial cell (iPSC‐EC) models to study angiogenesis in HGPS. We cultured normal and HGPS iPSC‐ECs under both static and fluidic culture conditions. HGPS iPSC‐ECs show reduced endothelial nitric oxide synthase (eNOS) expression and activity compared with normal controls and concomitant decreases in intracellular nitric oxide (NO) level, which result in deficits in capillary‐like microvascular network formation. Furthermore, the expression of matrix metalloproteinase 9 (MMP‐9) was reduced in HGPS iPSC‐ECs, while the expression of tissue inhibitor metalloproteinases 1 and 2 (TIMP1 and TIMP2) was upregulated relative to healthy controls. Finally, we used an adenine base editor (ABE7.10max‐VRQR) to correct the pathogenic c.1824C>T allele in HGPS iPSC‐ECs. Remarkably, ABE7.10max‐VRQR correction of the HGPS mutation significantly reduced progerin expression to a basal level, rescued nuclear blebbing, increased intracellular NO level, normalized the misregulated TIMPs, and restored angiogenic competence in HGPS iPSC‐ECs. Together, these results provide molecular insights of endothelial dysfunction in HGPS and suggest that ABE could be a promising therapeutic approach for correcting HGPS‐related cardiovascular phenotypes.

## Graphical abstract

Progerin antagonizes the normal angiogenic function via inhibiting endothelial nitric oxide synthase (eNOS) expression and activity and concomitant decrease in intracellular nitric oxide (NO) level.

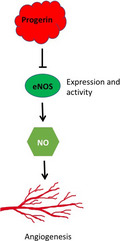

## 1 INTRODUCTION

Hutchinson–Gilford progeria syndrome (HGPS) is a rare, autosomal dominant disorder characterized by phenotypes reminiscent of premature aging (Burtner & Kennedy, 2010). The majority of HGPS cases are caused by a single *de novo* point mutation in *LMNA* (c.1824C>T; p.G608G) (Eriksson et al., 2003). This mutation activates a cryptic splice donor in exon 11 of the lamin A gene that results in mRNA mis‐splicing and that ultimately removes 150 nucleotides (50 amino acids) from the C‐terminal portion of the translated protein product. These 50 amino acids include an important proteolytic cleavage site that removes the farnesylated C‐terminus from prelamin A to form mature lamin A. Lack of this cleavage site results in the production of a persistently farnesylated, truncated lamin A protein variant called progerin (De Sandre‐Giovannoli et al., 2003; Eriksson et al., 2003). Progerin antagonizes normal lamin A function in a dominant‐negative manner, leading to nuclear abnormalities (Booth‐Gauthier et al., 2013; Goldman et al., 2004; Xiong et al., 2016), genomic instability (Gonzalo & Kreienkamp, 2015; Zhang, Sun, et al., 2016; Zhang et al., 2014), and altered redox homeostasis (Kubben et al., 2016; Xiong et al., 2017). Children with HGPS have clinical features that include short stature, prominent scalp vein, alopecia, loss of subcutaneous fat, osteoporosis, severe atherosclerosis, and accelerated organ degeneration (Ahmed et al., 2018; Merideth et al., 2008). Death results almost exclusively from coronary artery diseases or strokes at an average age of 14.6 years; currently, no cure exists for HGPS (Gordon et al., 2018; Harhouri et al., 2018).

The vascular endothelium plays a pivotal role in maintaining vascular homeostasis and tone by releasing various factors that regulate vascular smooth muscle cell function, inflammation, immune regulation, platelet aggregation, and thrombosis (Gimbrone & Garcia‐Cardena, 2016; Vanhoutte et al., 2017). One mechanism by which these processes are regulated by the endothelium is by activating endothelial nitric oxide synthase (eNOS), the enzyme responsible for nitric oxide (NO) production (Ignarro et al., 2001; Yang et al., 2009). Endothelial dysfunction disrupts vascular homeostasis and tone, leading to cardiovascular disease (Gimbrone & Garcia‐Cardena, 2016; Vanhoutte, 2009; Widmer & Lerman, 2014).

The role of progerin in endothelial dysfunctions and cardiovascular abnormalities has been explored *in vivo* and *in vitro*. Osmanagic‐Myers et al. generated VE‐cadherin promoter‐driven progerin in transgenic mice and showed early signs of diastolic dysfunction accompanied by cardiac hypertrophy, perivascular and interstitial fibrosis, and premature death (Osmanagic‐Myers et al., 2019). Similarly, Sun et al. generated Tie2‐driven progerin expression in a transgenic line and demonstrated endothelial dysfunction as a trigger of systemic aging (Sun et al., 2020). In addition, Dorado et al. showed vascular degenerations and cardiovascular complications in a knockin minipig model of HGPS (Dorado et al., 2019). We recently elucidated the HGPS iPSC‐ECs affect vascular functions in an *in vitro* tissue‐engineered blood vessel (TEBV) model of HGPS with increased inflammation markers (Atchison et al., 2020). These results provide strong evidence that progerin accumulation induces defects in endothelium and cardiovascular abnormalities.

One important process orchestrated by vascular endothelial cells is angiogenesis, the process of sprouting new blood vessels from the existing vasculature. Angiogenesis is a major adaptive response to physiological stress and is an endogenous repair mechanism following ischemic injury (Lahteenvuo & Rosenzweig, 2012; Potente & Carmeliet, 2017; Senger & Davis, 2011). Interestingly, angiogenic defects determined by reduced myocardial perfusion and decreased vascular density were observed in both knockin minipig and endothelium‐specific transgenic mouse models of HGPS (Dorado et al., 2019; Sun et al., 2020). The molecular mechanisms of progerin‐induced angiogenic incompetence are currently unknown.

This study investigates the underlying mechanisms of progerin‐induced disruption of angiogenesis. We measure endothelial deregulation in a well‐characterized iPSC‐derived EC model system, using eNOS expression and activity, intracellular NO level, and microvascular network formation as measures of angiogenesis potential of HGPS iPSC‐EC endothelial cells. These HGPS iPSC‐ECs express EC‐specific markers and manifest nuclear blebbing, a hallmark HGPS cellular phenotype (Atchison et al., 2020; Booth‐Gauthier et al., 2013; Goldman et al., 2004; Patsch et al., 2015). Using this system, we determine that an adenine base editor (ABE7.10max‐VRQR), which mediates the conversion of A•T‐to‐G•C in genomic DNA (Gaudelli et al., 2017; Koblan et al., 2018), rescues angiogenic phenotypes in HGPS iPSC‐ECs.

## 2 RESULTS

### 2.1 Characterization of human iPSC‐derived endothelial cells

**FIGURE 1:**
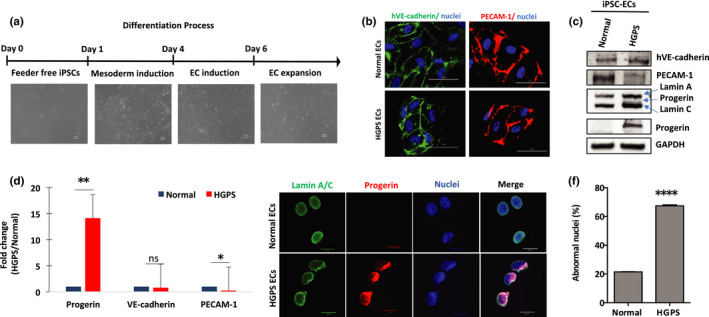
Characterization of normal and HGPS iPSC‐ECs. (a) Schematic illustration of the EC differentiation strategy from human iPSCs (scale bars = 100 μm). (b) Representative images of immunofluorescence staining of ECs with specific surface markers hVE‐cadherin and PECAM‐1 (scale bars = 50 μm). (c) Western blotting analysis with indicated antibodies on the cell lysates of normal and HGPS iPSC‐ECs. (d) Quantification of fold changes in the Western blot band densitometry in HGPS relative to the normal control. (e) Representative images of immunofluorescence staining with anti‐lamin A/C, anti‐progerin antibody, and DAPI on normal and HGPS iPSC‐ECs (scale bars = 20 μm). (f) Quantification of the percentage of abnormal nuclei in normal and HGPS iPSC‐ECs. Data are presented as mean ±SEM, *p < 0.05; **p < 0.01;****p < 0.0001; ns, not significant, *n*, three independent experiments; for nuclear morphology, *n* > 120 cells per group

Normal and HGPS endothelial cells (ECs) were differentiated from a pair of well‐characterized normal and HGPS iPSC lines (Figure 1a, Material Methods) (Atchison et al., 2017, 2020; Choi et al., 2018; Xiong et al., 2013; Zhang et al., 2014). Control and HGPS iPSC‐ECs both express human vascular endothelial cadherin (hVE‐cadherin) and platelet endothelial cell adhesion molecule‐1 (PECAM‐1, aka CD31), indicating consistent differentiation in both HGPS and normal controls (Figure 1b and Figure S1a). Progerin transcripts and protein were both abundant in HGPS iPSC‐ECs, leading to significant nuclear abnormalities compared with normal controls ten days post‐differentiation (Figure 1c‐f, Figure S1b‐c).

Confident that we had successfully differentiated HGPS iPSC‐ECs that manifest hallmark HGPS cellular properties, we next examined the endothelial characteristics of these cells. In normal endothelium, PECAM‐1 transmits mechanical force, while VE‐cadherin comprises the mechanosensory complex necessary for EC function and shear stress response (Tzima et al., 2005). Therefore, we examined PECAM‐1 and VE‐cadherin expression in HGPS iPSC‐ECs. In HGPS iPSC‐ECs, the transcript abundance of both PECAM‐1 (fourfold) and VE‐cadherin (CDH5) (threefold) was significantly reduced relative to normal controls (Figure S1a). The reduced transcript abundance of PECAM‐1 in HGPS iPSC‐ECs led to a fourfold decrease in PECAM‐1 protein abundance relative to healthy controls (Figure 1c,d), although VE‐cadherin protein abundance was comparable to healthy controls. Reduced PECAM‐1 transcript and protein abundance in HGPS iPSC‐ECs suggest a defect in sensing and responding to shear stress in these cells.

### 2.2 HGPS iPSC‐ECs exhibit functional defects in forming capillary‐like microvascular networks

**FIGURE 2:**
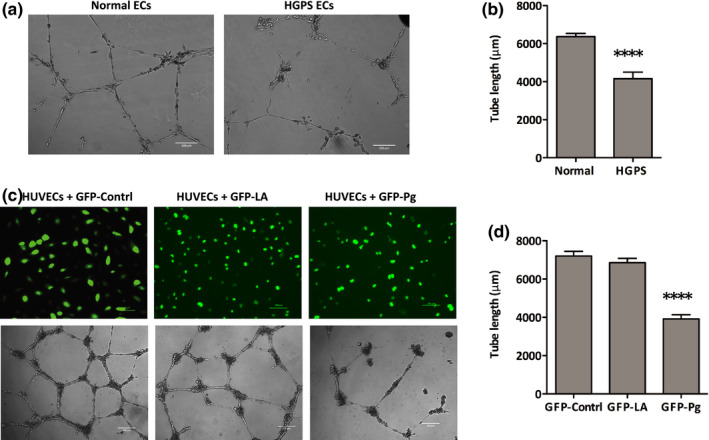
Capillary‐like microvascular network formation defects in HGPS iPSC‐ECs. (a) Matrigel‐based tube formation assay to assess the angiogenic activity of normal and HGPS iPSC‐ECs (scale bars = 200 μm). (b) Quantification of tube length per field. (c) Matrigel‐based tube formation assay to assess vascular network formation activity of HUVECs transduced with GFP‐control, GFP‐lamin A, or GFP‐progerin lentiviral vectors after 18 h (scale bars = 100 μm *top*, 200 μm *bottom*). (d) Quantification of tube length per field. ECs, endothelial cells; GFP, green fluorescent protein; LA, lamin A; Pg, progerin; HUVECs, human umbilical vein endothelial cells. Data are presented mean ±SEM, ***p < 0.0001; *n*, nine fields per group

Angiogenesis is a well‐established property of ECs (Lahteenvuo & Rosenzweig, 2012; Potente & Carmeliet, 2017; Senger & Davis, 2011). We hypothesized that progerin protein accumulation and consequential endothelial cell dysfunction would negatively impact angiogenesis in HGPS iPSC‐ECs. Indeed, phase‐contrast image analysis revealed that HGPS iPSC‐ECs form microvascular networks with significantly shorter capillary‐like structures than normal controls. HGPS microvascular tube structures average 4000 μm, significantly shorter than the average 6000 μm for normal controls (Figure 2a‐b). VE‐cadherin was aligned with the tube structures in both control and HGPS samples, suggesting appropriate tubular architecture was formed in both normal and HGPS iPSC‐ECs (Figure S2). To further characterize the angiogenic defect in HGPS iPSC‐ECs, we examined tube structure formation at different cell densities and time points. We observed shorter and fragmented capillary‐like tubes in HGPS iPSC‐ECs relative to healthy controls at all tested cell densities (10,000, 5000, and 2500 cells/well; Figure S3a). Time‐course studies further revealed that at a density of 5000 cells/well, normal and HGPS iPSC‐ECs first formed capillary‐like microvascular networks at 4 h post‐seeding. Microvascular networks generated by HGPS iPSC‐ECs began disintegrating at 8 h post‐seeding and shrank dramatically after 11 h, while the microvascular networks generated by normal cells were stable (Figure S3b and Supplemental Movies). These results indicate angiogenic defects in the HGPS iPSC‐ECs.

To confirm that angiogenic incompetence was directly induced by progerin, we used a lamin overexpression assay in human umbilical vein endothelial cells (HUVECs). HUVECs were transduced with lentiviruses expressing a GFP only control, a GFP–lamin A fusion protein, and a GFP–progerin fusion protein (Figure 2c). Progerin‐overexpressing HUVECs exhibited a significant reduction in capillary‐like microvascular network formation compared with the GFP–lamin A and GFP‐expressing controls after 18 h (Figure 2c‐d), confirming that progerin induces angiogenic defects in ECs.

### 2.3 HGPS iPSC‐ECs exhibit reduced total eNOS expression and activity

Having determined that progerin expression antagonizes microvascular network formation, we next sought to characterize how progerin expression alters eNOS function, a key regulator of angiogenesis, in ECs (Lu et al., 2015; Senger & Davis, 2011; Zhao et al., 2015). Besides its expression level, eNOS activity is typically controlled through protein–protein interactions and multisite phosphorylation events, especially phosphorylation at threonine 495 by protein kinase c suppresses its activity, both of which determine vascular density and NO production in the endothelium (Fukumura et al., 2001; Kolluru, Siamwala, et al., 2010; Kukreja & Xi, 2007; Mount et al., 2007; Yang et al., 2009).

**FIGURE 3:**
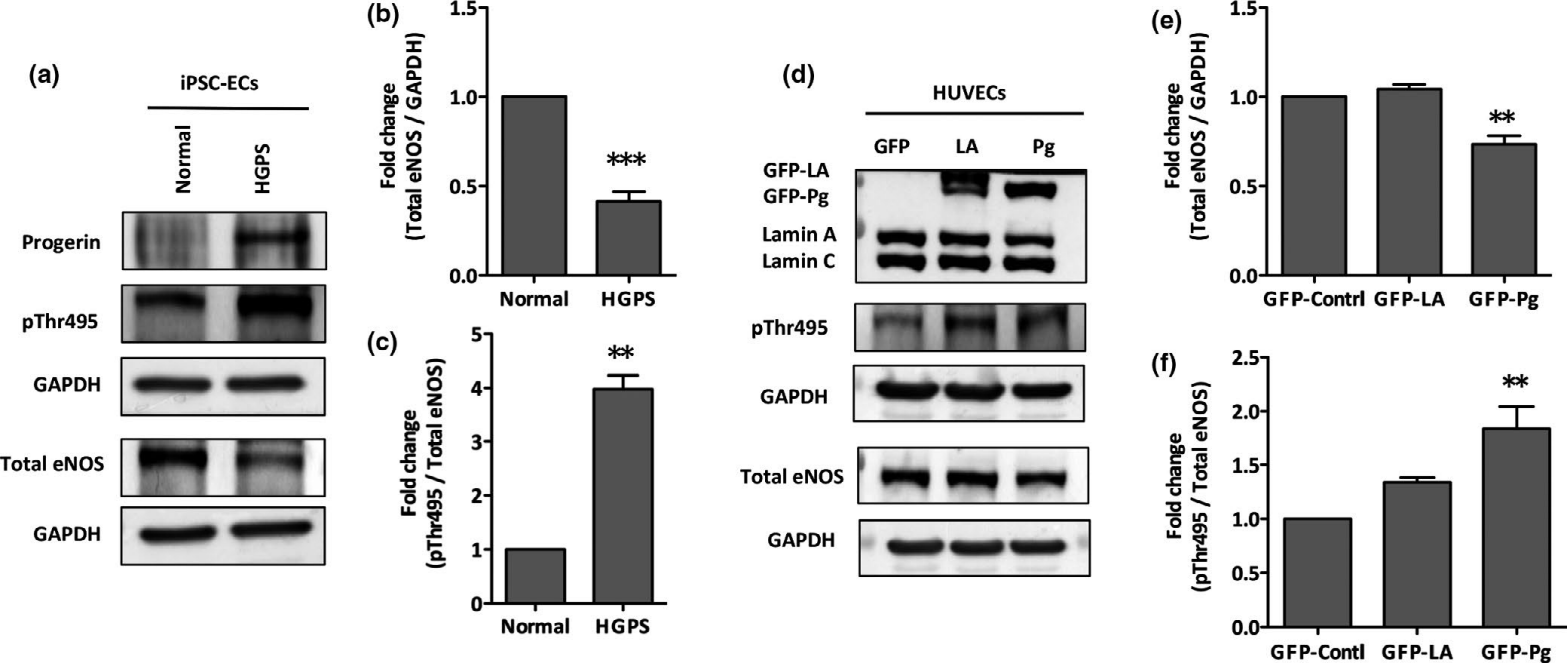
Downregulation of eNOS in HGPS iPSC‐ECs and in progerin‐overexpressed HUVECs. (a) Western blotting analysis with indicated antibodies on the lysates of normal and HGPS iPSC‐ECs. (b‐c) Quantification of fold change for Western blot band densitometry of total eNOS level normalized to GAPDH, and relative phosphorylated Thr495 level normalized to corresponding total eNOS, respectively. (d) Western blotting analysis with indicated antibodies on the lysates of HUVECs transduced with GFP‐control, GFP‐lamin A, or GFP‐progerin vectors. (e‐f) Quantification of fold change for Western blot band densitometry of total eNOS level normalized to GAPDH, and relative phosphorylated Thr495 level normalized to corresponding total eNOS, respectively. ECs, endothelial cells; eNOS, endothelial nitric oxide synthase; pThr495, phospho‐threonine 495; GFP, green fluorescent protein; LA, lamin A; Pg, progerin; HUVECs, human umbilical vein endothelial cells. Data are presented as mean ± SEM, **p < 0.01; ***p < 0.001, at least three independent replicates were performed in each experiment

Western blot analysis from lysates of normal and HGPS iPSC‐ECs revealed that total eNOS expression was twofold lower in HGPS iPSC‐ECs relative to normal controls (Figure 3a‐b). To assay eNOS function, we measured the abundance of phosphorylated threonine 495 eNOS, an inhibitory marker of eNOS activity (Fleming, 2010; Kolluru, Siamwala, et al., 2010). HGPS iPSC‐EC expression of phosphorylated threonine 495 eNOS was nearly fourfold higher than normal controls (Figure 3a,c). Similar results were observed in HUVECs transiently expressing progerin where total eNOS expression and activity were significantly lower than the healthy controls (Figure 3d‐f). These results establish that the presence of progerin reduces total eNOS abundance and activity in HGPS endothelial cells.

### 2.4 HGPS iPSC‐ECs are not able to upregulate eNOS in response to shear stress

**FIGURE 4:**
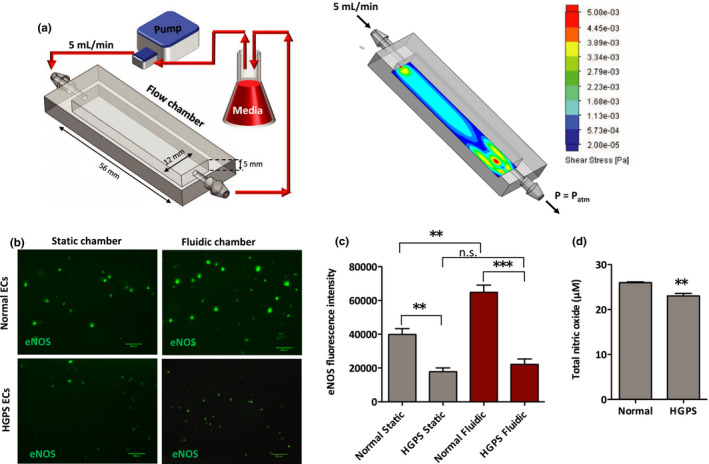
Inability to upregulate eNOS expression in HGPS iPSC‐ECs under shear stress. (a). Schematic illustration of the experimental setup of the fluidic culture chamber (*left*), and shear stress distribution on inner chamber surfaces from fluid flow simulation (*right*). (b) Immunofluorescence staining of eNOS in normal and HGPS iPSC‐ECs under static and fluidic culture conditions (scale bars = 200 μm). (c) Quantification of the eNOS fluorescence signal intensity. (d) Total intracellular NO level using a colorimetric assay in normal and HGPS ECs in the fluidic chamber. Data are presented as mean ± SEM, **p < 0.01; ***p < 0.001; *n*, three independent experiments

Shear stress stimulates eNOS expression and NO production (Boo et al., 2002; Cheng et al., 2005; Kolluru, Sinha, et al., 2010; Wang et al., 2010). Given that HGPS iPSC‐ECs show a fourfold reduction in PECAM‐1 expression (Figure 1c,d) and downregulated eNOS expression and activity (Figure 3a‐c), we hypothesized that the HGPS iPSC‐ECs might have defects in sensing shear stress. Therefore, we used a fluidic culture chamber with a flow of 5 mL/min to evaluate the functional properties of normal and HGPS iPSC‐ECs in response to shear stress. Fluidic culture chambers were fabricated using the EnvisionTEC Perfactory IV 3D printer, with E‐Shell^®^300 as the printing material, as reported in Lembong et al. (Lembong et al., 2018). Prior to cell seeding, flow chambers were coated with fibronectin. 250,000 normal or HGPS ECs were seeded per flow chamber. Theoretical shear stress profiles inside the flow chambers were calculated using the flow simulation feature of the computational modeling software SOLIDWORKS (Figure 4a). Up to 5 mPa was computed in the flow chamber, which is significantly below the reported shear stress value that causes mechanical damage and detachment of the ECs (Zhang, Liao, et al., 2016).

After seeding for 18, 24 h of shear stress was applied to iPSC‐ECs, which were then probed for eNOS expression. HGPS iPSC‐ECs show a significant reduction in total eNOS expression relative to normal controls under both static and fluidic conditions (Figure 4b,c). Normal iPSC‐ECs that experience applied sheer force (when cultured under fluidic conditions) show a significant induction of eNOS expression relative to cells cultured under static conditions. Remarkably, HGPS iPSC‐ECs show similar levels of eNOS expression under static and fluidic conditions (Figure 4b,c), resulting in a significant depletion of intracellular NO levels in HGPS iPSC‐ECs compared with normal controls under fluidic culture conditions (Figure 4d). Together, these results suggest that while eNOS expression is induced by shear stress in normal control ECs, HGPS iPSC‐ECs have defects in responding to the shear stress and therefore fail to upregulate eNOS expression.

### 2.5 HGPS iPSC‐EC angiogenic incompetence is eNOS‐dependent

**FIGURE 5:**
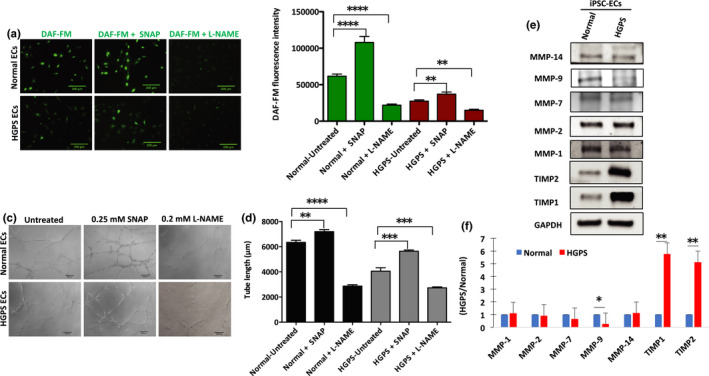
HGPS iPSC‐EC angiogenic incompetence is eNOS‐dependent. (a) Fluorescence images of NO, measured by DAF‐FM staining, were generated in normal and HGPS ECs either untreated or treated with 0.25 mM nitric oxide donor SNAP, and with 0.2 mM L‐NAME, an eNOS inhibitor (scale bars = 200 μm). (b) Quantification of the DAF‐FM fluorescence intensity for intracellular NO level in normal and HGPS iPSC‐ECs. (c) Matrigel‐based tube formation assay to assess the angiogenic activity of normal and HGPS iPSC‐ECs either untreated or treated with 0.25 mM SNAP, and with 0.2 mM L‐NAME (scale bars = 200 μm). (d) Quantification of tube length per field; *n*, nine fields per group. (e) Western blotting analysis with indicated antibodies on the lysates of normal and HGPS iPSC‐ECs. (f) Quantification of fold change for Western blot band densitometry of HGPS MMP and TIMP levels normalized to healthy control. DAF‐FM, 4‐amino‐5‐methylamino‐2’,7’‐difluorofluorescein diacetate; SNAP, S‐Nitroso‐N‐acetyl‐DL‐penicillamine; L‐NAME, N‐omega‐nitro‐L‐arginine methyl ester hydrochloride. Data are presented as mean ±SEM, *p < 0.05; **p < 0.01, ***p < 0.001, ****p < 0.0001; *n*, 3 independent experiments

We next sought to determine whether progerin‐induced eNOS deficiency is directly responsible for the angiogenic incompetence in HGPS iPSC‐ECs. To do so, we treated HGPS iPSC‐ECs and normal control ECs with an eNOS inhibitor, N‐omega‐Nitro‐L‐arginine methyl ester hydrochloride (L‐NAME), and a nitric oxide donor, S‐Nitroso‐N‐acetyl‐DL‐penicillamine (SNAP), and measured intracellular NO abundance. Fluorescence image analysis of NO, measured by 4‐amino‐5‐methylamino‐2’,7’‐difluorofluorescein diacetate (DAF‐FM) staining, was performed for both normal and HGPS iPSC‐ECs treated with SNAP or L‐NAME. Intracellular NO is significantly reduced in HGPS iPSC‐ECs compared with normal controls. SNAP treatment significantly increases intracellular NO at 0.25, 0.5, and 1 mM concentration in both normal and HGPS iPSC‐ECs (Figure S4). When normal ECs were treated with L‐NAME, however, intracellular NO levels were reduced to a similar level as in HGPS ECs (Figure 5a‐b; Figure S4). These results suggest that intracellular NO levels are reduced in HGPS iPSC‐ECs, likely due to impaired eNOS activity in these cells.

As described above, eNOS function and associated NO levels in cells affect endothelial angiogenic potential. We therefore examined the angiogenic competence of L‐NAME‐treated normal control ECs. Indeed, similar to HGPS iPSC‐ECs, normal iPSC‐ECs treated with L‐NAME show defective vascular network formation. In contrast, SNAP treatment at 0.25 mM and 0.5 mM concentration significantly increases the microvascular network formation, while at 1 mM concentration, it suppresses angiogenic efficiency of both normal and HGPS iPSC‐ECs (Figure 5c‐d; Figure S5a‐b). These results establish that eNOS deficiency is a key factor causing the angiogenic incompetence in HGPS iPSC‐ECs.

### 2.6 Upregulation of TIMP1 and TIMP2, and reduced expression of MMP‐9 in HGPS iPSC‐ECs

The matrix metalloproteinases (MMPs) and their specific inhibitors (tissue inhibitors of metalloproteinases; TIMPs) were recently described to be important regulators of angiogenesis (Brew et al., 2000; Davis & Senger, 2005; Ebrahem et al., 2011; Nagase et al., 2006; Saito et al., 2001). We therefore tested whether the expression profile of well‐established MMPs and their specific inhibitors differed in HGPS iPSC‐ECs relative to normal controls (MMP‐1, MMP‐2, MMP‐7, MMP‐9, MMP‐14, and their inhibitors TIMP1 and TIMP2) (Baker et al., 2002; Lee et al., 2010; Saito et al., 2001; Seo et al., 2003). Immunoblot analysis showed a sixfold increase in TIMP1 and a fivefold increase in TIMP2 in HGPS iPSC‐ECs relative to normal controls, while MMP‐9 (fourfold) was significantly reduced (Figure 5e). The elevated expression of TIMP1 and TIMP2 in combination with the suppressed expression of MMP‐9 in HGPS iPSC‐ECs likely limits the ability of these cells to degrade their extracellular matrix (ECM). Failure to degrade the ECM may suppress angiogenic VEGF signaling, possibly leading to the observed angiogenic incompetence in HGPS iPSC‐ECs.

### 2.7 **Adenine base editors (ABEs) efficiently correct the pathogenic HGPS mutation, rescue nuclear abnormalities, and increase angiogenic competence in HGPS iPSC‐ECs**.

**FIGURE 6:**
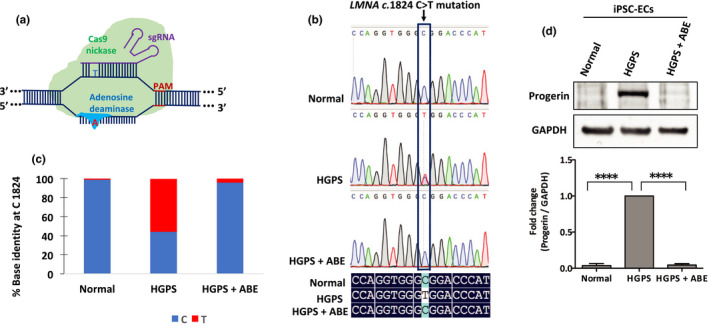
Correction of HGPS causing mutation by ABE in HGPS iPSC‐ECs. (a) Scheme of ABE‐mediated genome‐editing strategy. ABE targets the pathogenic adenosine (A) nucleobase in the human *LMNA* (c. 1824C>T) allele. A catalytically impaired Cas9 nickase–deoxyadenosine deaminase complex localizes to the *LMNA* gene locus by an allele‐specific sgRNA, generating a single‐stranded R‐loop of target genomic sequence. Exposed adenosines within the R‐loop can be deaminated by the fused evolved ssDNA‐specific deoxyadenosine deaminase domain. (b) Sanger sequence traces of normal, HGPS, and ABE‐edited HGPS iPSC‐ECs (top); sequence alignment of part of *LMNA* exon 11 from normal, HGPS, and ABE‐corrected HGPS ECs by DNAMAN software (bottom). (c) DNA sequence at the *LMNA* c.1824 nucleotide in normal, HGPS, and ABE7.10max‐VRQR lentivirus‐treated HGPS iPSC‐ECs after 20 days. (d) Western blotting analysis with indicated antibodies on the lysates of normal and HGPS iPSC‐ECs. Data are presented as mean ±SEM, ****p < 0.0001; *n*, three independent experiments

Hutchinson–Gilford progeria syndrome is caused by a single C>T point mutation in nuclear lamin A (*LMNA* c.1824 C>T) that can be corrected by an adenine base editor (ABE7.10max‐VRQR) programmed to correct the pathogenic c.1824 C>T mutation (Gaudelli et al., 2017; Koblan et al., 2018, 2021). ABE7.10max‐VRQR includes a catalytically impaired Cas9 D10A nickase programmed to recognize an NGA protospacer adjacent motif (PAM; VRQR Cas9) fused to an evolved single‐stranded DNA‐specific (ssDNA) deoxyadenosine deaminase that can be targeted to the pathogenic *LMNA* allele by specific single‐guide RNA (sgRNA) (Kleinstiver et al., 2016). Upon recognition of the appropriate PAM motif, unwinding of the double‐stranded DNA occurs as an RNA•DNA heteroduplex forms between the sgRNA spacer and the target DNA strand. Heteroduplex formation liberates a displaced single‐stranded segment of genomic DNA where some of this single‐stranded DNA, termed an R‐loop, is exposed outside of the Cas protein complex and is accessible to the ssDNA‐specific deoxyadenosine deaminase (Figure 6a) (Anzalone et al., 2020). The deoxyadenosine deaminase domain catalyzes the conversion of adenine to inosine, which is read as guanosine (G) by cellular polymerases. Through DNA repair and replication, the original A•T base pair is replaced with a G•C base pair at the target site (Gaudelli et al., 2017). The ABE‐based gene‐editing method is extensively studied in HGPS patient‐derived fibroblast cell lines and in an HGPS mouse model (Koblan et al., 2021).

**FIGURE 7:**
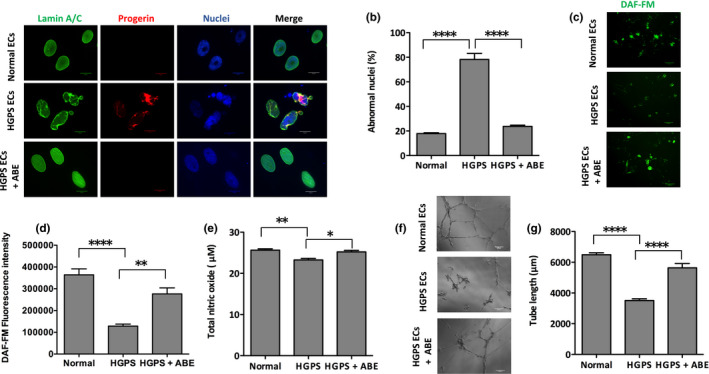
Rescue of the HGPS nuclear blebbing phenotype and increasing angiogenic competence in ABE‐corrected HGPS ECs. (a) Immunofluorescence staining with anti‐lamin A/C, anti‐progerin antibody, and DAPI on normal, HGPS, and ABE‐treated HGPS ECs (scale bars = 20 μm). (b) Quantification of the abnormal nuclei in normal, HGPS, and ABE‐treated HGPS ECs. (c) Fluorescence images of intracellular NO level, measured by DAF‐FM staining, in normal, HGPS, and ABE‐treated HGPS ECs (scale bars = 200 μm). (d) Quantification of the DAF‐FM fluorescence intensity for intracellular NO level in normal, HGPS, and ABE‐treated HGPS ECs. (e) Total NO bioavailability in normal, HGPS, and ABE‐corrected HGPS ECs using a colorimetric assay. (f) Matrigel‐based tube formation assay to assess the angiogenic activity of iPSC‐derived normal, HGPS, and ABE‐treated HGPS ECs (scale bars = 200 μm). (g) Quantification of tube length per field in (f). Data are presented as mean ± SEM, *p < 0.05; **p < 0.01; ****p < 0.0001; (*n*, three independent experiments; for nuclear morphology, *n* > 120 cells per group; for tube length, *n*, nine fields per group)

To test the efficacy of this approach in iPSC‐derived ECs, we cloned lentiviruses expressing ABE7.10max‐VRQR and either a c.1824 C>T correcting sgRNA or with control non‐targeting sgRNA. Following transduction of normal and HGPS iPSC‐ECs, we isolated and sequenced genomic DNA. We observed efficient correction of the HGPS mutation (*LMNA* c.1824 C>T) to the wild‐type sequence in cells treated with ABE and the c.1824 C>T correcting sgRNA with 96% correction at 20 days, but not in cells transduced with ABE and the non‐targeting sgRNA (Figure 6b,c). Indel frequencies were minimal (≤ 0.1%) for both HGPS and ABE‐corrected HGPS iPSC‐ECs. ABE‐mediated correction of the pathogenic allele reduced progerin expression levels by 95% (Figure 6d). Similarly, ABE‐treated HGPS iPSC‐ECs exhibit a significant reduction in progerin expression as determined by immunofluorescence analysis of fixed cells (Figure 7a).

We also examined the potential of ABE gene editing to rescue defects in both nuclear morphology and angiogenic potential of HGPS iPSC‐ECs. ABE treatment of the HGPS iPSC‐ECs reduced the number of misshapen nuclei by 54% relative to non‐targeting sgRNA control‐treated cells (Figure 7a,b). In addition, intracellular NO levels were restored to normal levels in ABE‐treated HGPS iPSC‐ECs (Figure 7c,d). Remarkably, ABE treatment increased iPSC‐EC capillary‐like tube length from 3510 µm in control sgRNA‐treated HGPS cells to 5638 µm in ABE‐corrected cells per field, which is comparable to normal control tube lengths (Figure 7e,f). Moreover, ABE treatment rescued the expression of MMP‐9, TIMP1, and TIMP2 toward the normal levels (Figure S6a, b). Together, the results establish that ABE‐mediated correction of the HGPS mutation reduces progerin protein abundance, rescues nuclear abnormalities, leads to increases in intracellular NO level, restores angiogenic competence, and normalized misregulated MMP‐9, TIMP1, and TIMP2 of HGPS iPSC‐ECs.

## 3 DISCUSSION

The underlying cause of death in most HGPS patients is cardiovascular failure. While it is well appreciated that endothelial dysfunction leads to cardiovascular remodeling and deregulated angiogenesis, very little is known about the mechanisms of progerin‐induced angiogenic incompetence in HGPS. Since HGPS is a very rare disease, and because the patients are very frail, it is challenging to obtain patient‐derived tissue‐specific cell lines to study HGPS disease mechanisms (Gordon et al., 2014). We have generated patient‐specific iPSCs to circumvent this challenge and developed an *in vitro* differentiation system to create a stable source of HGPS iPSC‐ECs (Figure 1). Our iPSC‐ECs express hVE‐cadherin and PECAM‐1 and form capillary‐like microvascular networks, exhibiting the vascular properties typical of mature ECs (Figure 2). Using this system, we characterized the progerin‐induced defects on capillary‐like microvascular network formation in HGPS iPSC‐ECs and elucidated the mechanisms responsible for this phenotype (Figure 5). We then use adenine base editing to directly correct the pathogenic allele and measure the consequences of genotypic correction on the vascular phenotypes of our iPSC‐EC model cells (Figures 6 and 7).

Genome‐wide expression profiling of HGPS fibroblasts revealed a widespread transcriptional misregulation leading to mesodermal defects and accelerated atherosclerosis (Csoka et al., 2004; McCord et al., 2013). In this regard, progerin deregulates myocardin‐related transcription factor‐A (MRTF‐A) that binds to the eNOS promoter and reduces eNOS expression (Osmanagic‐Myers et al., 2019). Progerin also induces oxidative stress in endothelial cells (Bidault et al., 2020), which can activate protein kinase C (PKC) that phosphorylates eNOS at threonine 495, leading to further inhibition of eNOS activity (Fleming, 2010; Mount et al., 2007). In agreement with these prior studies, here, we demonstrate that HGPS iPSC‐ECs show progerin‐dependent deficits in angiogenesis. We identify eNOS as a key regulator of these deficits where HGPS endothelial cells show reduced eNOS expression and activity and determine that progerin‐mediated deficiencies in eNOS function also impair responses to shear stress in HGPS iPSC‐ECs (Figure 4), likely driven by decreased expression of PCAM‐1 (Figure 1). Furthermore, we show that eNOS deregulation leads to depleted intracellular NO level under both static and fluidic culture conditions in HGPS iPSC‐ECs (Figure 4) and under static conditions in progerin overexpressing HUVEC HGPS model cells (Figure 3). It is tempting to speculate that the angiogenic incompetence of the HGPS ECs is related to the rarity of cancer in HGPS patients despite the high levels of DNA damage (Gonzalo & Kreienkamp, 2015; Kubben & Misteli, 2017; Musich & Zou, 2011). We also identified misregulated TIMP1, TIMP2, and MMP‐9 protein expression as a potential driver of progerin‐dependent angiogenic deficits in HGPS iPSC‐ECs (Figure 5). While these findings are correlative, the upregulation of TIMP1 and TIMP2 might provide a possible explanation of the massive depositions of collagen and fibronectin that result in fibrosis and hardening of the vessels in the cardiovasculature of children with HGPS. Future work is required to understand the roles of misregulated TIMP1, TIMP2, and MMP‐9 in HGPS pathogenesis. Finally, we use ABE7.10max‐VRQR to efficiently correct the HGPS mutation. ABE‐mediated genomic correction of the pathogenic allele reduces progerin protein abundance, rescues nuclear morphology similar in Ref. Koblan et al. (2021), increases the angiogenic competence, and normalizes the misregulated MMP‐9, TIMP1, and TIMP2 of corrected HGPS iPSC‐ECs (Figure 7; Figure S6).

Collectively, our findings show that HGPS leads to eNOS‐mediated angiogenic incompetence driven by reduced intracellular NO level and associated protein expression changes in metalloproteinases, TIMPs, and important cell surface proteins responsible for sensing shear stresses. We further show that ABE efficiently corrected HGPS mutation by rescuing the nuclear blebbing, normalizing misregulated MMP‐9, TIMP1, and TIMP2, and increasing the angiogenic competence of treated cells. Given that HGPS is a fatal premature aging disease, the improvement of angiogenic competence by this approach provides a promising path of intervention for clinical application. Moreover, minimal random insertion and deletion (indels) of the method offer feasible translational application. Despite its infancy, ABE could be a promising therapeutic approach for HGPS and other devastating monogenic diseases.

## 4 MATERIALS AND METHODS

### 4.1 Endothelial cell differentiation

For human iPSC differentiation, iPSC lines were derived from the normal father skin fibroblasts (HGADFN168) and HGPS patient (son) skin fibroblasts (HGADFN167) with the classic G608G HGPS mutation from Progeria Research Foundation Cell and Tissue Bank. EC differentiation was carried out using an *in vitro* monolayer endothelial differentiation of iPSC based on a previously established protocol with minor modifications in Refs Atchison et al. (2020) and Patsch et al. (2015). In brief, feeder‐free iPSCs were cultured in differentiation medium (DMEM/F12; Life Technologies, 11320–033), supplemented with Wnt agonist, glycogen synthase kinase 3 beta (GSK3β) inhibitor, CHIR‐99021, 5 μM (Cayman, 13122), bone morphogenetic protein‐4 (BMP4, 25 ng/ml) (PeproTech, 120‐05), B27 supplement (Life Technologies, 12587010), and N2 supplement (Life Technologies, 17502048) for three days. Then, to induce commitment to the endothelial lineage, cells were cultured in StemPro media (Life Technologies, 10639‐011) supplemented with forskolin, 5 μM (Abcam, ab120058), and vascular endothelial growth factor (VEGF165) 100 ng/ml (PeproTech, 100–20) for two days. iPSC‐ECs were then isolated by sorting on a FACS (BD) flow cytometer as described in Ref. Atchison et al. (2020) using a PE‐CD144‐conjugated antibody (mouse anti‐human; BD Pharmingen, 560411). Finally, cells were cultured in StemPro media supplemented with VEGF165 (50 ng/ml) and maintained at 37℃, and 5% CO_2_ in a humidified incubator. Cells were passaged once they reached 80–90% confluency.

### 4.2 Adenine base editor lentivirus

The ABE lentivirus construct was generated as described in Ref. Koblan et al. (2021). The human non‐targeting control sgRNA sequences are obtained from Doench et al. (2016). Oligos containing these non‐targeting sgRNAs with 5’ overhang BsmBI digestion sites were synthesized by Integrated DNA Technologies, Inc. The oligos were first annealed and inserted into the lentiCRISPR v2 plasmids (a gift from Feng Zhang, Addgene Plasmid #52961) as described previously in Sanjana et al. (2014). The fragments containing these non‐targeting sgRNAs were digested from the recombinant lentiCRISPR v2 plasmids by restriction enzymes KpnI and NheI. These fragments were then ligated into the ABEmax7.10 backbone, which was extracted from the digests of KpnI and NheI. The sequences of the recombinant plasmids were confirmed by Sanger sequencing.

### 4.3 Fabrication of fluidic chambers

Fluidic culture chambers were designed in SOLIDWORKS (Dassault Systems), as described by Lembong et al. (2018) with minor modifications. The inner culture chamber was 56 × 12 × 5 mm in length, breadth, and height with no internal geometries that could hinder fluid flow. The chambers were 3D‐printed using the EnvisionTEC Perfactory IV 3D printer and E‐Shell^®^300 as the resin material. Following printing, chambers were washed in isopropanol to eliminate residual, uncross‐linked material, and then flash‐cured. For sterilization, chambers were filled with ethanol and then exposed to ultraviolet light for an hour. Chambers were then rehydrated by serial washes in the following sterile solutions: (1) 75% ethanol/25% phosphate‐buffered saline (PBS), (2) 50% ethanol/50% PBS, (3) 25% ethanol/75% PBS, and (4) 100% PBS, and then stored in PBS. Before cell seeding, flow chambers were coated with 3 µg/cm^2^ fibronectin (Corning, 356008) in PBS for 1h at 37℃ to facilitate cell attachment. Chambers were connected to a peristaltic pump set to 5 ml/min with media circulation.

### 4.4 Immunocytochemistry

For immunocytochemistry, cells were washed once with PBS and fixed in 4% paraformaldehyde (PFA) for 15 min. Cells were then blocked with 10% normal donkey serum in PBS (blocking buffer) for 1 h. When probing for an intracellular antigen, 0.3% Triton‐X was included in the blocking buffer. Cells were then incubated with the primary antibodies lamin A/C (MAB3211; Millipore, 1:250), progerin (Cao et al., 2011) with a dilution of 1: 250, goat anti‐VE‐cadherin (AF938; R&D Systems, 1:100), and sheep anti‐PECAM‐1 (AF806, R&D Systems, 1:100), in 5% normal donkey serum in PBS overnight at 4°C. After three washes with PBS, cells were incubated in 1% BSA in PBS‐containing secondary antibodies and DAPI (Vector Laboratories). Secondary antibodies used for immunocytochemistry were Alexa Fluor^®^ 594 donkey anti‐rabbit IgG (Invitrogen, 1:1000) and Alexa Fluor^®^ 488 donkey anti‐mouse IgG (Invitrogen, 1:1000 dilution). Cells were washed three times with PBS, and images were acquired with Zeiss AX10 microscope equipped with a SPOT PURSUIT camera. For the analysis of nuclear morphology, greater than 120 nuclei were analyzed per condition where the nuclei were assigned by visual inspection into normal or abnormal nuclear phenotype.

### 4.5 Western blotting

Whole cell lysates for immunoblotting were prepared by dissolving cells in Laemmli sample buffer containing 5% of 2‐mercaptoethanol (Bio‐Rad). Primary antibodies used for immunoblotting include anti‐lamin A/C (MAB3211; Millipore, 1:500 dilution), progerin (Cao et al., 2011) with a dilution of 1:500, anti‐eNOS (D9A5L; Cell Signaling, 1:1000), anti‐pThr eNOS (Cell Signaling, 1:1000), mouse anti‐GAPDH (sc‐47724; Santa Cruz, 1:3000), goat anti‐VE‐cadherin (AF938; R&D Systems, 1:500), and anti‐PECAM‐1 (AF806, R&D Systems, 1:500), anti‐MMP‐1, MMP‐2, MMP‐7, MMP‐9, MMP‐14, TIMP1, and TIMP2 (Kit # 73959, Cell Signaling, 1:1000 dilution). Secondary antibodies include anti‐mouse (sc‐516102; Santa Cruz, 1:5000), anti‐rabbit (211–035–109; Jackson Immuno‐Research, 1:5000), anti‐goat (HAF017; R&D Systems, 1:1000), and anti‐goat (HAF019; R&D Systems, 1:1000 dilution).

### 4.6 RNA extraction, cDNA synthesis, and RT‐qPCR

Total RNA from human cell lines was extracted with TRIzol Reagent (Life Technologies, 15596026) and purified using the RNeasy Mini Kit (Qiagen) as per the manufacturer's instructions. RNA yield was determined using the NanoDrop 2000 Spectrophotometer (Thermo Scientific). Total RNA (1μg) was converted to cDNA using the iScript Select cDNA Synthesis Kit (Bio‐Rad) according to manufacturer's instructions. Quantitative RT‐PCR was carried out in triplicate using SYBR Green Supermix (Bio‐Rad), and detection was achieved using CFX96 Real‐Time System (C1000 Thermal Cycler, Bio‐Rad). Gene expression was calculated as a relative fold change using the ΔCt method of analysis and normalized to GAPDH. The specific primer sequences are described in Table 1.

**TABLE 1:** Primer sequences used for RT‐PCR experiment

Target gene	Primer sequence
GAPDH	Forward: 5′‐GTCTCCTCTGACTTCAACAGCG‐3′
	Reverse: 5′‐ACCACCCTGTTGCTGTAGCCAA‐3′
CDH5	Forward:5′‐GAAGCCTCTGATTGGCACAGTG‐3′
	Reverse: 5′‐TTTTGTGACTCGGAAGAACTGGC‐3′
PECAM‐1	Forward: 5′‐AAGTGGAGTCCAGCCGCATATC‐3′
	Reverse: 5′‐ATGGAGCAGGACAGGTTCAGTC‐3′
LMNA	Forward: 5′‐GCAACAAGTCCAATGAGGACCA‐3′
	Reverse: 5′‐CATGATGCTGCAGTTCTGGGGGCTCTGGAT‐3′
Progerin	Forward: 5′‐GCAACAAGTCCAATGAGGACCA‐3′
	Reverse: 5′‐CATGATGCTGCAGTTCTGGGGGCTCTGGAC‐3′

### 4.7 Nitric oxide assays

After 48 h of incubation, 1 ml media was taken and flash‐frozen in liquid nitrogen and frozen at −80°C. For the cell in the fluidic bioreactors, after 24 h of perfusion, 1 ml media was removed from the flow circuit and flash‐frozen in liquid nitrogen and frozen at −80°C. Prior to testing, samples were spun at 9500 RCF for 7 min with a 10,000 MWCO spin column (Corning). Total nitrate and nitrite were measured using a colorimetric assay (Arbor Assays) as per kit instructions. Absorbance was measured at 540 nm using a 96‐well microplate reader. For the second method of intracellular NO level measurement, we used DAF‐FM (Thermo Scientific). iPSC‐ECs were either treated with 0, 0.25, 0.5, and 1 mM nitric oxide donor S‐Nitroso‐N‐acetyl‐DL‐penicillamine (SNAP, N3398; Sigma‐Aldrich), or with an eNOS inhibitor (0.2 mM N‐omega‐nitro‐L‐arginine methyl ester hydrochloride; L‐NAME, N5751; Sigma‐Aldrich), and then incubated with DAF‐FM 10 μM at 37°C, and 5% CO_2_ for 30 min as per manufacturer's recommendation. Excess probe was removed by washing with PBS, and the cells were switched to fresh media prior to imaging. Images were acquired with Zeiss AX10 microscope equipped with a SPOT PURSUIT camera. The intensity of the DAF‐FM fluorescent signal (intracellular NO level) was measured using ImageJ and plotted as mean of the fluorescent signal (integrated density) in each cell.

### 4.8 Tube formation assay

A 150 µl of Matrigel (Corning Matrigel Basement Membrane Matrix Growth Factor Reduced, Phenol Red Free, 356231) was aliquoted to 48‐well plates and incubated for 30 min at 37℃ to allow the gel to solidify. 20,000 iPSC‐ECs were seeded onto the Matrigel matrix and cultured for 18 h at 37℃, and images were acquired using Zeiss AX10 microscope equipped with a SPOT PURSUIT camera. The tube length was measured using ImageJ software. For the cell density and time‐course experiment in Figure S3, the specified cells were seeded on 96‐well plate using 75 µl of Matrigel. To test the angiogenic potentials of the live normal and HGPS iPSC‐ECs, 10,000 cells were seeded on 96‐well plate with iPSC‐EC media. Then, spinning disk confocal images were acquired for 18 h using an UltraVIEW VoX System (PerkinElmer, Waltham, MA) attached to an inverted microscope (Eclipse Ti; Nikon Instruments, Melville, NY) with a 10× NA 1.3 objective (Nikon) and a CCD camera (C9100‐50; Hamamatsu Photonics, Bridgewater, NJ) with maintaining the culture condition of 37℃, and 5% CO_2_.

### 4.9 Statistical analysis

Statistical analyses were performed using GraphPad Prism 7 software. Data were evaluated using Student's unpaired *t test* for two groups, and one‐way analysis of variance (ANOVA), and a post hoc Tukey test to compare means of three or more groups. Results are presented as the mean ± SEM. *p* value < 0.05 was considered significant.

## CONFLICTS OF INTEREST

D.R.L. is a consultant and co‐founder of Editas Medicine, Pairwise Plants, Beam Therapeutics, and Prime Medicine, companies that use genome‐editing technologies. [Correction added on 11 June 2021, after first online publication: Conflict of interest text has been modified in this version].

## AUTHOR CONTRIBUTIONS

Y.G.G. and K.C designed the project. Y.G.G., L.W.K., X.M., M.T., and B.M performed the experiments. K.C, J.P.F., and D.R.L provided reagents. Y.G.G. and K.C wrote and edited the manuscript with coauthor contributions. All authors reviewed the manuscript and provided critical analysis of the manuscript.

## Supporting information

Supplementary Material

Video S1

Video S2

**Fig. S1:** (A). RT‐qPCR analysis of endothelial‐specific marker genes, CDH5 and PECAM‐1. Their relative mRNA levels were normalized to GAPDH. (B). RT‐qPCR analysis of *LMNA* and progerin in normal and HGPS iPSC‐ECs. (C). Representative image of immunofluorescence staining with anti‐lamin A/C, anti‐progerin antibody, and DAPI on normal and HGPS iPSC‐ECs (scale bars = 100 µm). Mean standard deviation, Students unpaired *t‐test*; *p < 0.05; **p < 0.01; ***p < 0.001; ns = not significant; n= 3 independent experiments
**Fig. S2:** Images of VE‐cadherin staining on the capillary‐like vascular networks formed by normal or HGPS iPSC‐ECs at 18 hours. Scale bars = 200 µm.
**Fig. S3:** (A). Phase contrast images of tube structures formed by normal or HGPS iPSC‐ECs at indicated cell densities after 18 hours. (B). Phase contrast images of tube structures formed by normal and HGPS iPSC‐ECs at the cell density of 5000 per well during a time‐course experiment (scale bars = 200 µm).
**Fig. S4:** (A). Fluorescence images of NO, measured by DAF‐FM staining, were generated in normal and HGPS ECs either untreated or treated with o.25, 0.5 and 1 mM nitric oxide donor SNAP, and with 0.2 mM L‐NAME, an eNOS inhibitor (scale bars = 200 µm). (B). Quantification of the DAF‐FM fluorescence intensity for intracellular NO level in normal and HGPS iPSC‐ECs. DAF‐FM, 4‐Amino‐5‐Methylammino‐2’,7’‐Difluorofluerescene Diacetate; SNAP, S‐Nitroso‐N‐acetyl‐DL‐penicillamine; L‐NAME, N‐omega‐Nitro‐L‐arginine methyl ester hydrochloride.
**Fig. S5:** (A). Matrigel‐based tube formation assay to assess the angiogenic activity of normal and HGPS iPSC‐ECs either untreated or treated with 0.25, 0.5 , and 1 mM nitric oxide donor SNAP, and with 0.2 mM L‐NAME (scale bars = 200 µm). (B). Quantification of tube length per field. DAF‐FM, 4‐Amino‐5‐Methylammino‐2’,7’‐Difluorofluerescene Diacetate; SNAP, S‐Nitroso‐N‐acetyl‐DL‐penicillamine; L‐NAME, N‐omega‐Nitro‐L‐arginine methyl ester hydrochloride. Data are presented as mean ± SEM, p < 0.05,⭑***p < 0.001, ****p < 0.0001; n= 9 fields per group.
**Fig. S6:** (A). Western blot analysis with indicated antibodies on the lysates of normal, HGPS and ABE treated HGPS iPSC‐ECs. (B). Quantification of fold change for western blot band densitometry of MMP‐9, TIMP1 and TIMP2 levels normalized to healthy control. Data are presented as mean ± SEM, *p < 0.05; **p < 0.01; n= 3 independent experiments.
**Table 1:** Primer sequences used for RT‐PCR experiment.

## Data Availability

Data sharing not applicable—no new data generated.
