# Supplementary material for: Mechanisms of angiogenic incompetence in Hutchinson–Gilford progeria via downregulation of endothelial NOS
Source: Aging Cell. 2021 Jun 4;20(7):e13388. doi: 10.1111/acel.13388 (PMC8282277; doi:10.1111/acel.13388)
Supplement: Supplementary file 1 — Supplementary Material [file ACEL-20-e13388-s001.pdf]

Supplemental Figure-1

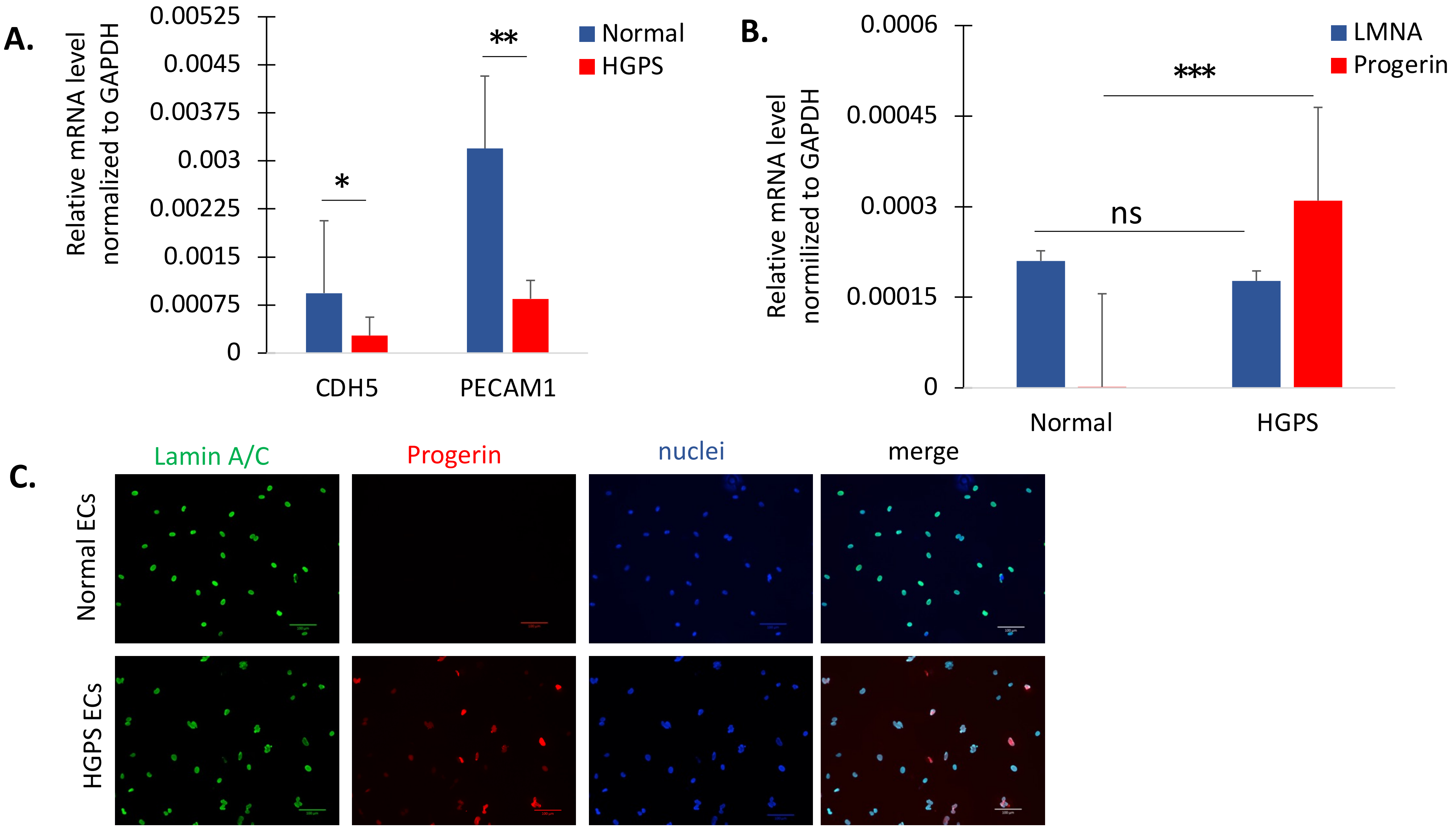

Supplemental Figure-2

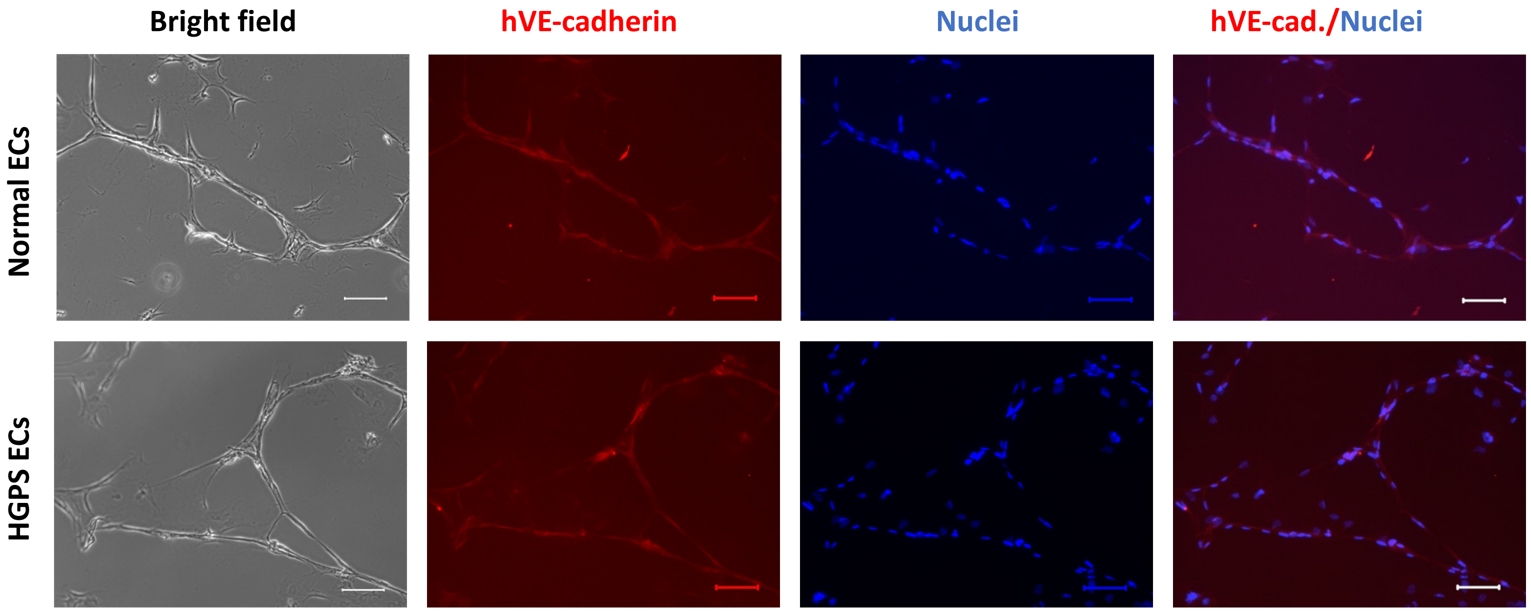

Supplemental Figure-3

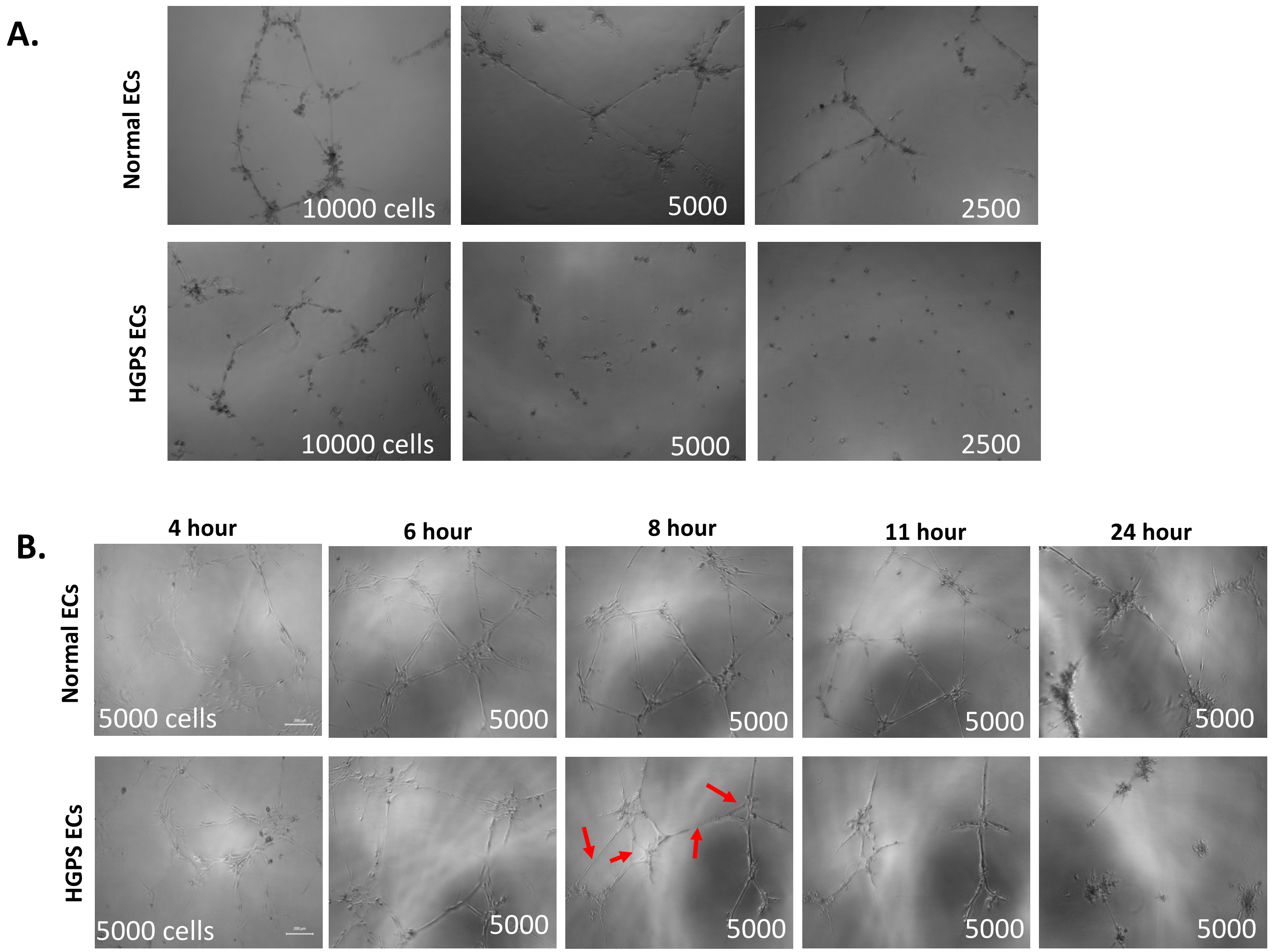

Supplemental Figure-4

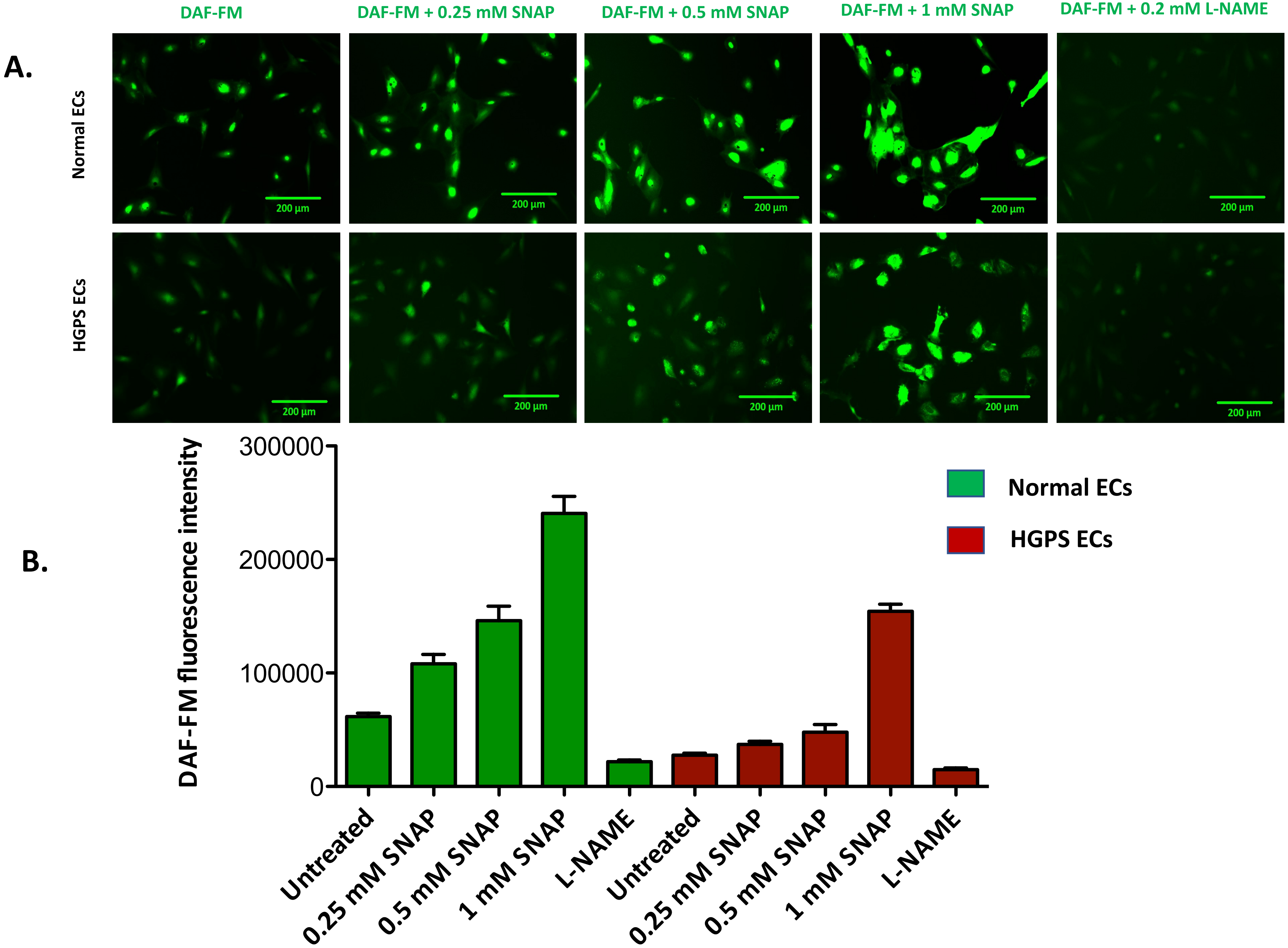

Supplemental Figure-5

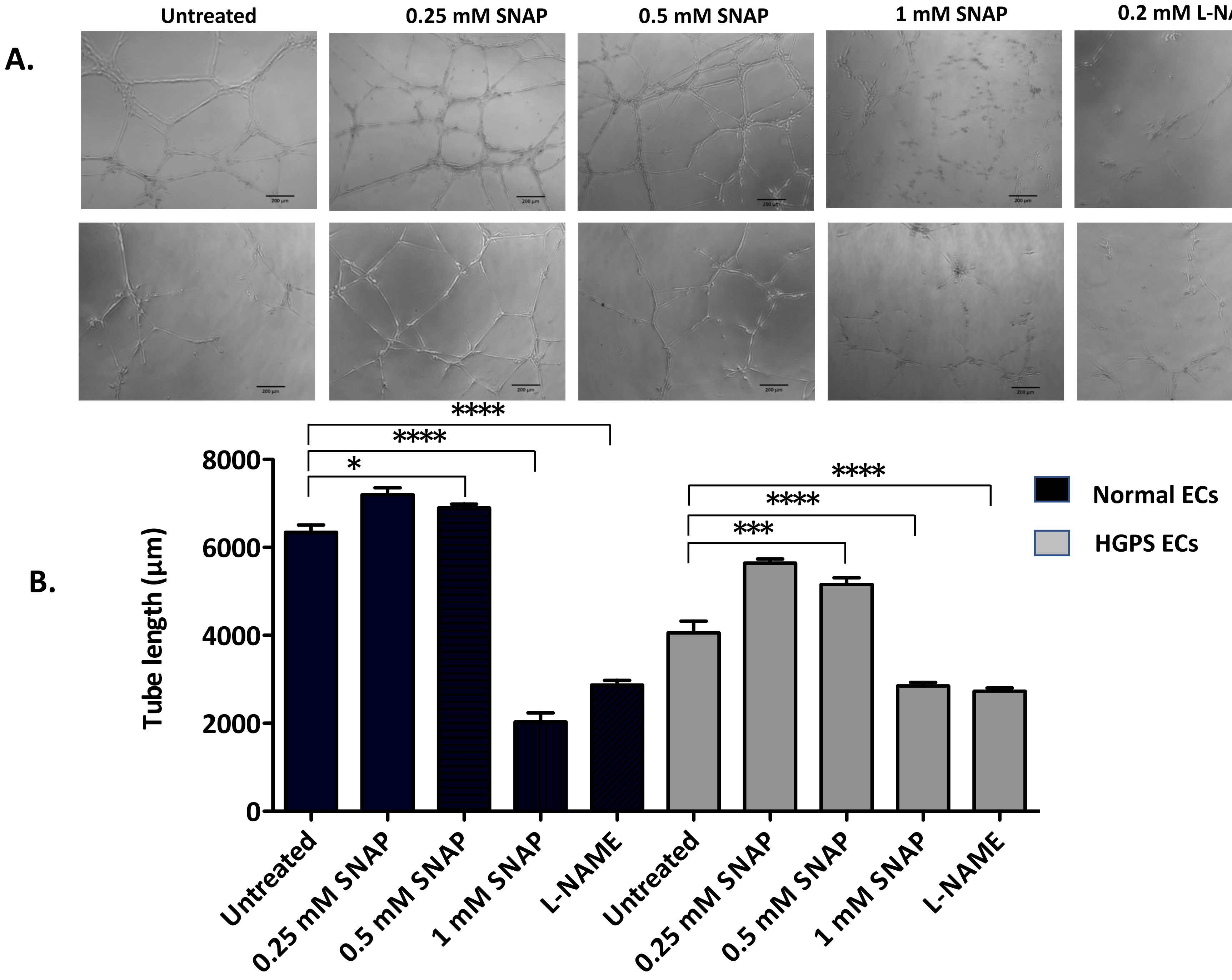

Supplemental Figure-6

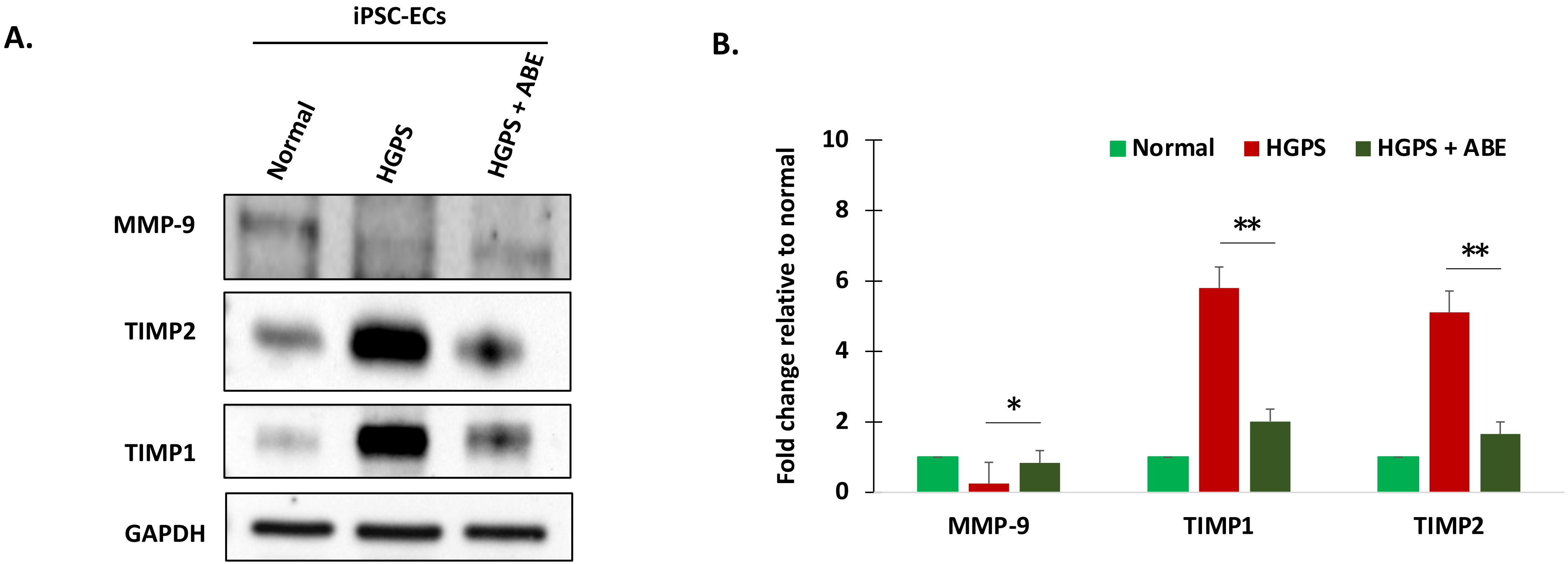

Table 1: Primer sequences used for RT-PCR experiment

Target gene	Primer sequence
GAPDH	Forward: 5'-GTCTCCTCTGACTTCAACAGCG-3'
	Reverse: 5'-ACCACCCTGTTGCTGTAGCCAA-3'
CDH5	Forward:5'-GAAGCCTCTGATTGGCACAGTG-3'
	Reverse: 5'-TTTTGTGACTCGGAAGAACTGGC-3'
PECAM-1	Forward: 5'-AAGTGGAGTCCAGCCGCATATC-3'
	Reverse: 5'-ATGGAGCAGGACAGGTTCAGTC-3'
LMNA	Forward: 5'-GCAACAAGTCCAATGAGGACCA-3'
	Reverse: 5'-CATGATGCTGCAGTTCTGGGGGCTCTGGAT-3'
progerin	Forward: 5'-GCAACAAGTCCAATGAGGACCA-3'
	Reverse: 5'-CATGATGCTGCAGTTCTGGGGGCTCTGGAC-3'
