# Supplementary material for: Mechanisms of angiogenic incompetence in Hutchinson–Gilford progeria via downregulation of endothelial NOS
Source: Aging Cell. 2021 Jun 4;20(7):e13388. doi: 10.1111/acel.13388 (PMC8282277; doi:10.1111/acel.13388)
Supplement: Supplementary file 4 — Fig. S1: (A). RT‐qPCR analysis of endothelial‐specific marker genes, CDH5 and PECAM‐1. Their relative mRNA levels were normalized to GAPDH. (B). RT‐qPCR analysis of LMNA and progerin in normal and HGPS iPSC‐ECs. (C). Representative image of immunofluorescence staining with anti‐lamin A/C, anti‐progerin antibody, and DAPI on normal and HGPS iPSC‐ECs (scale bars = 100 µm). Mean standard deviation, Students unpaired t‐test; *p < 0.05; **p < 0.01; ***p < 0.001; ns = not significant; n= 3 independent experiments Fig. S2: Images of VE‐cadherin staining on the capillary‐like vascular networks formed by normal or HGPS iPSC‐ECs at 18 hours. Scale bars = 200 µm. Fig. S3: (A). Phase contrast images of tube structures formed by normal or HGPS iPSC‐ECs at indicated cell densities after 18 hours. (B). Phase contrast images of tube structures formed by normal and HGPS iPSC‐ECs at the cell density of 5000 per well during a time‐course experiment (scale bars = 200 µm). Fig. S4: (A). Fluorescence images of NO, measured by DAF‐FM staining, were generated in normal and HGPS ECs either untreated or treated with o.25, 0.5 and 1 mM nitric oxide donor SNAP, and with 0.2 mM L‐NAME, an eNOS inhibitor (scale bars = 200 µm). (B). Quantification of the DAF‐FM fluorescence intensity for intracellular NO level in normal and HGPS iPSC‐ECs. DAF‐FM, 4‐Amino‐5‐Methylammino‐2’,7’‐Difluorofluerescene Diacetate; SNAP, S‐Nitroso‐N‐acetyl‐DL‐penicillamine; L‐NAME, N‐omega‐Nitro‐L‐arginine methyl ester hydrochloride. Fig. S5: (A). Matrigel‐based tube formation assay to assess the angiogenic activity of normal and HGPS iPSC‐ECs either untreated or treated with 0.25, 0.5 , and 1 mM nitric oxide donor SNAP, and with 0.2 mM L‐NAME (scale bars = 200 µm). (B). Quantification of tube length per field. DAF‐FM, 4‐Amino‐5‐Methylammino‐2’,7’‐Difluorofluerescene Diacetate; SNAP, S‐Nitroso‐N‐acetyl‐DL‐penicillamine; L‐NAME, N‐omega‐Nitro‐L‐arginine methyl ester hydrochloride. Data are presented as mean ± SEM, p < 0.05, [file ACEL-20-e13388-s002.docx]

**Supplementary information**

**Mechanisms of angiogenic incompetence in Hutchinson-Gilford Progeria via downregulation of endothelial NOS**

Yantenew G. Gete^1^, Luke W. Koblan^2,3,4^, Xiaojing Mao^1^, Mason Trappio^1^, Bhushan Mahadik^5^, John P. Fisher^5^, David R. Liu^2,3,4^, Kan Cao^1,*^

1. Department of Cell Biology and Molecular Genetics, University of Maryland, College Park, MD 20742, USA.

2. Merkin Institute of Transformative Technologies in Healthcare, Broad Institute of Harvard and MIT, Cambridge, MA, USA.

3. Department of Chemistry and Chemical Biology, Harvard University, Cambridge, MA, USA.

4. Howard Hughes Medical Institute, Harvard University, Cambridge, MA, USA.

5. Fischell Department of Bioengineering, University of Maryland, College Park, MD 20742, USA.

**Supplemental Figure Legends**

**Fig. S1:** (A). RT-qPCR analysis of endothelial-specific marker genes, CDH5 and PECAM-1. Their relative mRNA levels were normalized to GAPDH. (B). RT-qPCR analysis of *LMNA* and progerin in normal and HGPS iPSC-ECs. (C). Representative image of immunofluorescence staining with anti-lamin A/C, anti-progerin antibody, and DAPI on normal and HGPS iPSC-ECs (scale bars = 100 µm). Mean standard deviation, Students unpaired *t-test*; *p < 0.05; **p < 0.01; ***p < 0.001; ns = not significant ; n= 3 independent experiments.

**Fig. S2:** Images of VE-cadherin staining on the capillary-like vascular networks formed by normal or HGPS iPSC-ECs at 18 hours. Scale bars = 200 µm.

**Fig. S3:** (A). Phase contrast images of tube structures formed by normal or HGPS iPSC-ECs at indicated cell densities after 18 hours. (B). Phase contrast images of tube structures formed by normal and HGPS iPSC-ECs at the cell density of 5000 per well during a time-course experiment (scale bars = 200 µm).

**Fig. S4:** (A). Fluorescence images of NO, measured by DAF-FM staining, were generated in normal and HGPS ECs either untreated or treated with o.25, 0.5 and 1 mM nitric oxide donor SNAP, and with 0.2 mM L-NAME, an eNOS inhibitor (scale bars = 200 μm). (B). Quantification of the DAF-FM fluorescence intensity for intracellular NO level in normal and HGPS iPSC-ECs. DAF-FM, 4-Amino-5-Methylammino-2’,7’-Difluorofluerescene Diacetate; SNAP, S-Nitroso-N-acetyl-DL-penicillamine; L-NAME, N-omega-Nitro-L-arginine methyl ester hydrochloride.

**Fig. S5:** (A). Matrigel-based tube formation assay to assess the angiogenic activity of normal and HGPS iPSC-ECs either untreated or treated with 0.25, 0.5 , and 1 mM nitric oxide donor SNAP, and with 0.2 mM L-NAME (scale bars = 200 μm). (B). Quantification of tube length per field. DAF-FM, 4-Amino-5-Methylammino-2’,7’-Difluorofluerescene Diacetate; SNAP, S-Nitroso-N-acetyl-DL-penicillamine; L-NAME, N-omega-Nitro-L-arginine methyl ester hydrochloride. Data are presented as mean ± SEM, **p < 0.05,⭑*****p < 0.001, ****p < 0.0001; n= 9 fields per group.

**Fig. S6:** (A). Western blot analysis with indicated antibodies on the lysates of normal, HGPS and ABE treated HGPS iPSC-ECs. (B). Quantification of fold change for western blot band densitometry of MMP-9, TIMP1 and TIMP2 levels normalized to healthy control. Data are presented as mean ± SEM, *p < 0.05; **p < 0.01; n= 3 independent experiments.

Table 1: Primer sequences used for RT-PCR experiment.
